# Integrated targeted and non-targeted analysis of water sample extracts with micro-scale UHPLC–MS

**DOI:** 10.1016/j.mex.2015.10.002

**Published:** 2015-10-14

**Authors:** Dominik Deyerling, Karl-Werner Schramm

**Affiliations:** a TUM, Wissenschaftszentrum Weihenstephan für Ernährung, Landnutzung und Umwelt, Lehrstuhl für analytische Lebensmittelchemie, Alte Akademie 10, 85354 Freising, Germany; b Helmholtz Zentrum München – Deutsches Forschungszentrum für Gesundheit und Umwelt, Molecular EXposomics, Ingolstädter Landstr. 1, 85764 Neuherberg, Germany; c TUM, Wissenschaftszentrum Weihenstephan für Ernährung, Landnutzung und Umwelt, Department für Biowissenschaften, Weihenstephaner Steig 23, 85350 Freising, Germany

**Keywords:** Universal UHPLC–MS analysis enhanced by non-target screening, UHPLC–MS, Non-target, Pharmaceutical residues, Pesticides, Extraction, Cleanup, Retention time index, Database research

## Abstract

A sensitive method is introduced to detect selected pharmaceutical residues and polar pesticides with UHPLC–MS in water samples of different origin. Active or passive water sampling was combined with a laboratory solid-phase extraction cleanup and stable isotope dilution analysis. Recovery experiments demonstrated that the internal standard correction performed well for the compensation of matrix effects. Besides, the original targeted analysis approach was expanded by non-target analysis of the samples with only one more consecutive injection run needed. The key benefits of this multi-residue analysis are:

• Targeted analysis and quantification combined with non-target analysis on a micro-scale UHPLC–MS system usually employed for qualitative analysis only.
• The internal standards for targeted analysis were used in non-target runs to calculate the partition coefficient log *P* of unknown substances employing the retention time index (RTI).
• The filtering of database hits for two criteria (exact mass and partition coefficient) significantly reduced the list of suspects and at the same time rendered it possible to perform non-target analysis with lower mass accuracy (no lock-spray) in the range of 20–500 ppm.

## Graphical abstract

**Figure:**
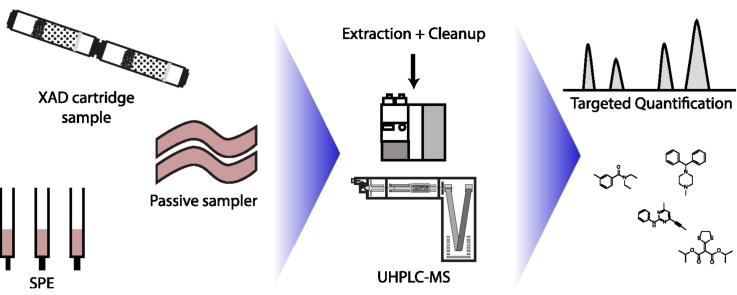

## Method details

The analysis of organic trace compounds in water is a challenging task requiring analyte enrichment in most analytical approaches. For the analysis of polycyclic aromatic hydrocarbons (PAHs) we routinely applied passive samplers to which we refer to as virtual organisms [1], [2], [3]. In a more recent study, passive sampling was accompanied by the active on-site enrichment of PAHs on self-packed glass cartridges with polymeric resin [4]. This active sampling technique allowed us to cover a wider range of more polar analytes by the variation of the employed polymeric resin. Therefore, we developed a new instrumental approach allowing the analysis of samples from different sample origin toward a more polar group of analytes like pharmaceutical residues and polar herbicides. Method development for some selected target compounds as well as for non-target analysis was carried out on a nano-scale UHPLC–MS system coupled to a Q-TOF mass spectrometer. The nano-scale chromatography system, commonly applied in proteomic approaches, promises detection with high sensitivity and less matrix suppression which are abilities of which also environmental analysis could benefit. With operating the chromatography system with a micro-scale analytical column, we could further enlarge the application spectrum of this universal instrument.

## Materials and instrumentation

The list of selected target compounds for instrumental method development is presented in Table 1. Sulfamethoxazole and naproxen were obtained as native standard substances from Dr. Ehrenstorfer (Augsburg, Germany, now part of the LGC group, Teddington, UK), simazin, atrazine and linuron were obtained from Riedl-de Haën (Seelze, Germany) and carbamazepine from Sigma–Aldrich (Steinheim, Germany). For the preparation of a stock solution from the native compounds, a precision balance SBC 21 from SCALTEC Instruments GmbH (Goettingen, Germany) was used. The isotopically labeled equivalents, sulfamethoxazole-D4, carbamazepine-D10, naproxen-D3, simazin-D5, atrazine-D5 and linuron-D6 were purchased from Dr. Ehrenstorfer already dissolved at 100 ng μL^−1^ in either acetonitrile, methanol or acetone. Acetonitrile and methanol were obtained from Promochem (Wesel, Germany) in UHPLC–MS grade. Ultrapure water was generated with a water purification system consisting of a RiOs reverse osmosis unit with Progard 1 silver cartridge and a Milli-Q Gradient unit with Quantum EX Ultrapure Organex cartridge + Q-Guard 1, both from Merck Millipore (Darmstadt, Germany). Polypropylene solid-phase extraction cartridges packed with 500 mg polar modified polystyrene–divinylbenzene copolymer Chromabond HR-X were obtained from Macherey-Nagel (Dueren, Germany). Polymeric resins Amberlite XAD7HP and XAD16N for self-packed water sampling glass cartridges was purchased from Sigma–Aldrich. Prior usage, sampling cartridges packed with these resins have been extracted in a Soxhlet apparatus with 800 mL of acetone for a period of at least 16 h.

The analytical instrument consisted of a nanoAcquity ultra high-performance liquid chromatography (Waters, Massachusetts) coupled to a quadrupole time-of-flight mass spectrometer (Q-TOF2, Waters-Micromass, UK). The chromatography was coupled via a standard electrospray interface to the mass spectrometer at a flow rate of 4 μL min^−1^. The separation was carried out in micro-scale on a HSS-T3 C-18 reversed phase column (150 mm × 300 μm i.d., 1.8 μm particle size, Waters). In general, micro-scale separation in combination with the standard electrospray interface granted higher stability and reproducibility than a corresponding setup with a nano flowrate of 0.3 μL min^−1^. Gradient elution was established with a binary gradient pump setup with solvent A (0.1% formic acid in water) and solvent B (0.1% formic acid in acetonitrile). The gradient is shown in Table 2. The autosampler with conditioned sample compartment (10 °C) was equipped with a 5 μL sample loop and required 10 μL of sample per injection (loop overfill). The column temperature was maintained at 30 °C. The TOF mass calibration was renewed on a daily basis by direct infusion of either 0.1% phosphoric acid in acetonitrile:water 50:50 (v:v) or 1 mM sodium formate in 90:10 2-propanol:water (v:v). The capillary voltage optimized at 2.5 kV. Nitrogen at a flow rate of 200 L h^−1^ was used as desolvation gas at a temperature of 120 °C. The ion source block itself was heated to 100 °C. Argon 5.0 was used as collision gas. The cone voltage and collision energy were acquired for each analyte separately by directly infusing native stock solutions of the single compounds in acetonitrile at a concentration of 1 ng μL^−1^. The optimized parameters can be found in Table 3. Analyte quantification was carried out in TOF-MS mode passing a broad range of ions into the collision cell as the time-of-flight detection allowed us to detect all ion fragments simultaneously and at the same time increasing the sensitivity. This setting equals the ‘fragment all ions’ option of more modern devices. Due to limitations of the mass spectrometer, the collision energy in TOF-MS mode could only be set to a mean value rather than being adjusted to the optimum fragmentation energy of each compound during the analytical run. Nevertheless, the gains in sensitivity overcompensated the lower or higher degree of fragmentation caused by the mean collision energy set.

## Water sample origin

Details of the water sampling campaigns have already been described elsewhere and are not the topic of this work [1], [2], [4]. Nevertheless, suitable sample origins tested with the presented method shall be briefly mentioned here. Surface water with low analyte concentrations expected was enriched on-site with self-packed glass cartridges with a dimension of 22 cm × 4.5 cm (length × diameter). The cartridges were filled with 50:50 mixture of Supelpak XAD7/16. The resin was trapped within the cartridges with a glass frit at one side and heat-treated glass wool at the other side. For sampling, two cartridges (master and backup) were submerged in water at the sampling site and a water volume of about 300 L was sucked through the cartridges at a flow rate of about 2 L min^−1^ with a battery-driven peristaltic pump. The volume was monitored with an analog water meter. Depending on the sampling location, a filter glass cartridge, tightly packed with heat-treated glass wool was mounted in front of the sampling cartridges to exclude large particulates from entering the sampling cartridges. With the high enriched sample volume and the corresponding high enrichment factor, theoretical environmental concentrations between 30 and 300 pg L^−1^ could be determined.

Passive water samples were gathered with virtual organisms (SPMD-like passive samplers) which consist of 25 mm wide and 65 μm thick lay-flat polyethylene tubing (VWR, Ismaning, Germany), cut to a length of 29 cm and filled with 700 μL of triolein (Sigma, Munich, Germany). The triolein was trapped within the tubing by heat-seals at both ends. For exposure, the samplers were mounted in stainless steel cages, submerged in about 1 m water depth. Typical exposure time was between 7 and 14 days. In case of polycyclic aromatic hydrocarbons (PAHs) the accumulation mechanism of these samplers from the water phase is already well understood and can be tracked by the dissipation of isotopically labeled performance reference compounds spiked into the triolein during the production of the passive sampler [5], [6], [7]. In case of the presented method development, dialysates of exposed samplers were investigated with targeted and non-targeted analysis.

Finally, water samples taken with 1 L glass bottles have been analyzed by direct solid-phase extraction of 250 mL water that was directly guided through the SPE cartridge.

## Extraction and clean-up

From the XAD sampling cartridges excess of water was removed prior extraction by a gentle stream of nitrogen for 5 min. Subsequently, the cartridges were spiked with an internal standard mixture including all investigated analytical targets as mass-labeled compounds. Afterwards, the cartridges (with still slightly wetted polymeric resin) were transferred to a soxhlet apparatus and extracted with 800 mL acetone during 16 h overnight which corresponded to about 60 extraction cycles. On the following day, the extracts were evaporated by rotary evaporation to a volume of about 200 mL. Remaining water was removed from the extract by directing the extract through glass filters filled with dry sodium sulfate. Finally, the dried extract was evaporated to a volume of about 2 mL and ready for the following cleanup step with solid-phase extraction.

The VOs were transferred for dialysis into 250 mL Erlenmeyer flasks filled with 100 mL acetonitrile:methanol 50:50 (v:v) and gently shaken with an orbital shaker for 16 h overnight. After dialysis, the VO was removed and the dialysate was spiked with mass labeled standards and evaporated to a volume of about 2 mL, ready for SPE cleanup.

The solid-phase extraction was carried out on a vacuum manifold. The elution protocol was kept equal for each sampling source, except for direct solid-phase extraction of water:

1. Conditioning with 5 mL acetonitrile:methanol 50:50 (v:v) followed by 5 mL ultrapure water.
2. Sample transfer to SPE cartridge; 3× rinsing of sample flask with about 0.2 mL ultrapure water; in case of direct-solid phase extraction: 250 mL water sample, spiked with mass-labeled standards, administered to the cartridge with a flow rate of 4 mL min^−1^.
3. Washing with 5 mL ultrapure water.
4. Drying for about 40 min under gentle vacuum.
5. Sample elution with 2 mL × 5 mL acetonitrile:methanol 50:50 (v:v) into pear shaped flask.

The eluate was evaporated by rotary evaporation to a volume of about 200 μL and transferred to a 1.5 mL polypropylene tube by three times rinsing with acetonitrile:methanol 50:50 (v:v). Finally, a sample volume of typically 200 μL was adjusted by evaporating excess solvent under a gentle stream of nitrogen and heating to 40 °C.

## Calibration and cleanup performance

**Fig. 1:**
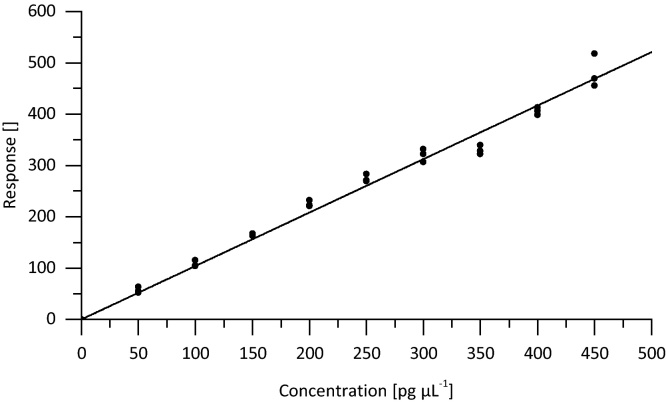
Internal standard corrected calibration curve of atrazine 0–450 pg μL^−1^; *R*^2^ = 0.9958; *y* = 1.042*x*; *n* = 3.

From the native stock solution with all compounds dissolved in acetonitrile at 100 ng μL^−1^, dilutions for a 10-point calibration were prepared in 90:10 solvent A:B (v:v) mobile phase with concentrations of 0, 50, 100, 150, 200, 250, 300, 350, 400 and 450 pg μL^−1^. The concentration of mass labeled compounds was kept constant in each calibration solution at 400 pg μL^−1^. The peak areas determined for the native compounds were corrected for the signal intensity of the corresponding co-eluting internal standard substance. The plot of the calibration curve represents the linear regression of internal standard (IS) corrected response against the standard concentration. In terms of a verification experiment, the standards were measured in triplicate on a monthly basis (see Fig. 1 for atrazine). Besides, prior to each sample analysis a calibration curve was issued by single injections of the 10-point calibration standards.

**Fig. 2:**
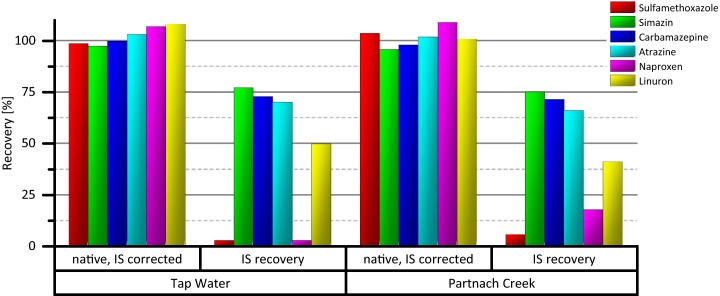
Internal standard corrected recovery of native compounds in tap water and surface water spiked with known amounts of analytical targets; the IS recovery is an indicator for signal suppression due to matrix effects.

The performance of the HR-X cartridges for analyte enrichment was determined with water samples of clean sources in terms of the investigated analytes. The samples were analyzed by the previously described direct solid-phase extraction of 250 mL volume. Both samples (tap water and surface water sampled at the spring of Partnach creek, Germany) were analyzed but no analyte signal could be detected. Thus, the performance of the HR-X cleanup could be evaluated with the remaining water samples by spiking native standard mixture and mass-labeled standards. The results are illustrated in Fig. 2. Internal standard corrected recoveries are above 90% for all analytes in both samples. The recovery of internal standards is an indicator for apparent matrix effects. In both samples, severe signal suppression as matrix effect could be observed for sulfamethoxazole and naproxen, partly below 10% IS recovery. Besides, linuron seems to be affected with suppression of the IS signal between 50% and 59%. For the majority of investigated compounds, signal suppression is higher in the surface water sample. The results demonstrate good performance of the isotope dilution technique in the compensation of matrix effects.

## Non-target analysis

The described analysis for targeted compounds was extended with a non-target analysis. Therefore, the samples were injected subsequently after targeted analysis. The cone voltage was set to 25 V and the collision energy to 5 V to minimize ion fragmentation and to thus detect positively charged molecular ions. The non-target injections were accompanied by blank injections in order to correct the non-target runs for background noise. Sample runs already acquired for targeted analysis with elevated collision energy were used at the same time to screen for characteristic ion fragments of promising suspects. The signals of the mass-labeled internal standards were used to calibrate the chromatographic system with the retention time index (RTI) [8], [9] in order to calculate values for the partition coefficient of unknown signals based on their retention time. The retention time index is basically a normalization of the log *P* value on a linear scale from 50 to 150. The first and last eluting compounds were set to 50 and 150, respectively. Once the boundaries were set, the RTI was calculated according to the following equation:

$${\text{RTI}}_{i}=(\log\;P_{i}-\log\;P_{i-1})\cdot \left[\frac{150-50}{\log\;P_{\max}-\log\;P_{\min}}\right]+{\text{RTI}}_{i-1}$$

where the index *i* indicates the compounds in eluting order, log *P*_max_ and log *P*_min_ are the maximum and minimum values of the partition coefficient within the calibration, respectively (usually the first and last eluting compounds).

**Fig. 3:**
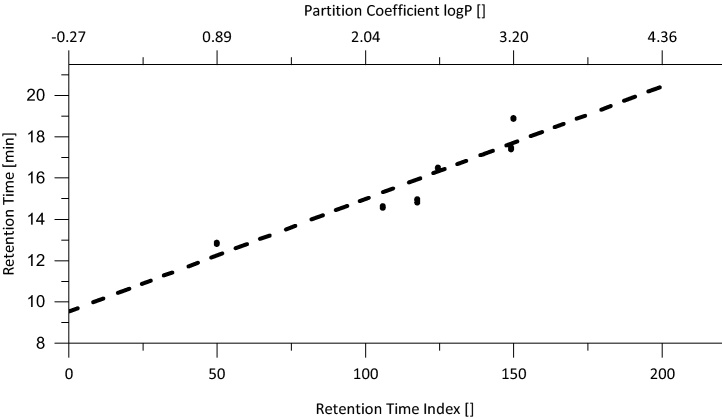
Correlation between retention time and retention time index (normalized partition coefficient) gathered from calibration standard injections for targeted analysis (*n* = 9); linear regression is shown as dashed line, *R*^2^ = 0.85583.

The RTI is linearly correlated with the retention time (Fig. 3). The log *P* value of unknown suspects was gathered by first calculating their corresponding RTI by linear interpolation between the two corresponding standards. Subsequently, the RTI was converted to the log *P* scale again. For compounds eluting earlier or later than the calibrated targeted compounds, the linear correlation was used to estimate the corresponding log *P* value.

The complete workflow of non-target analysis for one sample involved the following steps:

1. Subtraction of the signal of a blank injection measured prior to the non-target sample in MassLynx 4.1.
2. Conversion of the blank corrected MS data to NetCDF exchange format with DataBridge-tool for further processing in MZmime 2.14.
3. Cropping of irrelevant parts of the chromatogram including early elution and re-equilibration.
4. Generation of a peak list from the total ion chromatogram for signals with a minimum peak height of 50 counts per second.
5. Separation of multiple peaks with the single chromatograms into separate rows of the peak list (deconvolution).
6. Removal of duplicates due to isotope patterns with an isotopic pattern filter.
7. Visible judgment of suspect list and removal of chromatograms which appear to be noise.
8. Calculation of partition coefficient based on the retention time of the suspects.
9. Suspect identification with the databases STOFF-IDENT, DAIOS and MassBank using exact mass and the calculated partition coefficient as first identifiers and detected mass fragments as verifiers.

The non-target workflow was developed with water samples gathered with virtual organisms exposed in the small river Selke which is a tributary of the river Bode in Saxony-Anhalt (Germany). In total, two exposed samples, one field blank and one laboratory blank were investigated. Targeted analysis could not detect investigated compounds in blanks as well as in the exposed samples. The concurrent non-target analysis ended up with suspect lists of 15 and 20 possible suspects, respectively. The main criterion for exclusion was the signal intensity. The alignment of the sample lists from both exposed samples indicated only two suspects existing in both samples which can be regarded as the most promising ones. One of these substances could be identified as *N*,*N*-diethyl-*m*-toluamide (DEET) with high likelihood which is a common ingredient in insect repellents. The calculated exact mass of the single positive charged molecular ion of this compound is 192.1388 *m*/*z*, the acquired values were 192.1343 *m*/*z* and 192.1464 *m*/*z* in the two independent analyzed samples. This result equals a mass deviation between 23 and 40 ppm which is an acceptable value for the applied mass spectrometry without lock-mass correction. The mass accuracy, however, is too low to allow a reasonable prediction of the elemental formula from the exact mass. The log *P* value for this substance was determined with the RTI calibration to 2.70 and 2.74 which equals only a deviation of 0.2 to the value 2.50 predicted by algorithm of ChemAxon. Additionally, the partition coefficient indicates that the substance has a low solubility in water, which promotes the enrichment within the passive samplers. The polarity of the suspect as well as its phenyl core structure is similar to the compounds investigated in targeted analysis, thus, making it feasible that the compound may be enriched during the SPE cleanup, too. Besides, the structural similarity of this suspect to the compounds from the targeted approach assures the analytical availability during chromatography and ionization. Fragmentation information on DEET obtained by LC–MS was available in DAIOS and MassBank. In total, three characteristic mass fragments of DEET (119.05, 91.06 and 72.05) could be found at its retention time during the targeted analysis run (MS run with elevated collision energy). All in all, the results gave strong evidence, that DEET was present in both passive samplers. Finally, the comparison of the analyte signal with a standard prepared from the purchased native substance DEET confirmed the suspect. Further suspects and their verification level are presented in Table 4.

The developed non-target workflow was used to process water samples established with the cartridge sampling method described above. The samples were taken in the Yangtze River slightly upstream of the Three Gorges Dam (China) at Maoping. Samples of two water depths (31 and 50 m) were investigated with non-target analysis, each consisting of a master and backup cartridge. Therefore, the abundance pattern of signals within the four investigated sampling cartridges was used as selection criterion for possible suspects. Only suspects with the following pattern have been considered:

• Detectable in all four sampling cartridges.
• Detectable in the master and backup cartridge of either 31 or 50 m water depth.
• Detectable in both master or both backup cartridges from 31 and 50 m water depth.

This abundance pattern excludes the possible case that a suspect would be detectable in a single master or backup cartridge within one water depth. However, the probability for this case can be regarded as low. Substances which are within the polarity range enriched by the employed XAD-resin, should usually be detectable either in the master cartridge or in master and backup cartridge. Consequently, the employed abundance pattern only excludes substances from the analytical results which were only detectable within one sampling depth.

The described suspect abundance criterion helped to reduce the suspect list from 96 to 23 entries. For further 7 suspects, database research revealed no possible structures or structures which would have been unlikely to be enriched with the employed sampling method or unavailability to LC–MS. Further 8 suspects could not be verified due to the lack of MS fractionation data in MassBank. Four structures with a match in terms of exact mass and polarity (log *P*-value) had to be rejected due to a mismatch in MS fragments. Finally, the 4 remaining suspects could be verified with at least 1 matched MS/MS fragment in MassBank (see Table 5). Again, DEET was found and verified with a corresponding native standard substance. Additionally, the fungicides isoprothiolane and mepanipyrim, commonly applied in agriculture for the protection of rice, wine, strawberries, tomatoes and cucumber [10], could be verified with corresponding mass fragments. In the case of isoprothiolane, the sulfur atoms were also reflected within the recorded isotopic pattern. The last suspect cyclizine, a first-generation antihistamine, could only be verified with one MS/MS fragment.

## Additional information

The recent advances in analytical sciences made it possible to detect a wide spectrum of organics of anthropogenic origin in the environment. The prescription of and expenditures on pharmaceuticals in Germany rose continuously during the last 10 years and thereby their consumption [11]. The active ingredients are usually only partly metabolized [12]. The excreted pharmaceutical residues could be frequently detected in surface water as they often survive wastewater treatment [13], [14], [15]. At the same time, the environmental effects of these pharmaceutically active compounds are not known and can only be estimated [16]. Therefore, there is a demand for a sensitive and reliable analytical method for the detection of these pharmaceutical ingredients. The presented method for targeted analysis was proven suitable for the sensitive detection of the investigated analytes in water. Particularly, the stable isotope dilution analysis performed well in correcting the results for the strong matrix effects observed. Besides, it was possible to enhance the targeted method with a non-target approach although lacking of a mass detection with high accuracy. We could demonstrate that the shortcoming regarding mass accuracy of the mass spectrometer could be compensated by taking selectivity of sample origin, cleanup and measurement parameters combined with chemical knowledge into account. As this approach is available for transfer to other MS applications with low mass accuracy or lower mass resolution. The amount of suspects was kept low by blank correction and signal intensity restriction. The remaining suspects were finally checked with database research. Therefore, the partition coefficient as additional search criterion complementing the exact mass further reduced possible hits.

## Figures and Tables

**Table 1:** List of compounds, sorted by eluting order, investigated in targeted analysis; log *P* values were calculated with the algorithm from ChemAxon.

Compound name	Classification	Molecular formula	Partition coefficient log *P* [–]	Exact mass M + H [Da]
Sulfamethoxazole	Antibiotic	C_10_H_11_N_3_O_3_S	0.79	254.05993
Carbamazepine	Anticonvulsant	C_15_H_12_N_2_O	2.45	237.10279
Simazin	Herbicide	C_7_H_12_ClN_5_	2.18	202.08595
Atrazine	Herbicide	C_8_H_14_ClN_5_	2.61	216.10160
Naproxen	NSAID	C_14_H_14_O_3_	3.18	231.10212
Linuron	Herbicide	C_9_H_10_Cl_2_N_2_O_2_	3.20	249.01976

**Table 2:** Binary chromatographic gradient; isocratic hold at injection start was needed to flush the 5 μL sample loop at the analytical flow rate of 4 μL min^−1^.

Time [min]	Solvent A [%]	Solvent B [%]
00.0	100	0
01.5	100	0
25.0	0	100
30.0	0	100
30.1	100	0
35.0	100	0

**Table 3:** Optimized mass spectrometer parameters for targeted analysis and quantification.

Compound name	Cone voltage [V]	Collision energy no fragmentation [V]	Collision energy MS/MS [V]
Sulfamethoxazole	25	5	16
Carbamazepine	40	5	19
Simazin	30	5	18
Atrazine	30	5	18
Naproxen	30	5	18
Linuron	30	5	15

**Table 4:** List of possibly identified suspects in the investigated passive samplers and their verification level regarding *m*/*z*, log *P*, MS/MS fragments and blank control.

Suspect *m*/*z*	Possible compound	Δ*m*/*z* [ppm]	Δlog *P* [–]	Amount MS/MS fragments	Found in Blank	Found in both samples
192.1404	*N*,*N*-Diethyl-*m*-toluamide (DEET)	32	+0.20	3	No	Yes
214.0827	Salicylanilide	19	−0.42	2	Yes	No
185.1498	γ/δ-Undecalactone	24	+0.74	3	No	No
283.2529	Artemisinin	347	−0.08	0	No	No

**Table 5:** List of possible suspects with name, structure, *m*/*z* deviation, log *P* deviation from predicted ChemAxon log *P*-value and abundance pattern.

Possible compound	Chemical structure	Δ*m*/*z* [ppm]	Δlog *P* [–]	Detected in 31 m master + backup/51 m master + backup/field blank [+/−]
*N*,*N*-Diethyl-*m*-toluamide		360	+0.11	+|+|+|+|−

Isoprothiolane		348	+0.87	+|+|+|+|−

Mepanipyrim		238	+0.52	+|+|+|+|−

Cyclizine		316	+1.07	+|−|+|+|−
